# Nation-Wide Routinely Collected Health Datasets in China: A Scoping Review

**DOI:** 10.3389/phrs.2022.1605025

**Published:** 2022-09-21

**Authors:** Yishu Liu, Shaoming Xiao, Xuejun Yin, Pei Gao, Jing Wu, Shangzhi Xiong, Carinna Hockham, Thomas Hone, Jason H. Y. Wu, Sallie Anne Pearson, Bruce Neal, Maoyi Tian

**Affiliations:** ^1^ George Institute for Global Health, University of New South Wales, Newtown, NSW, Australia; ^2^ The George Institute for Global Health, Health Science Centre, Peking University, Beijing, China; ^3^ School of Public Health, Health Science Center, Peking University, Beijing, China; ^4^ National Center for Chronic and Non-Communicable Diseases Control and Prevention, Chinese Center for Disease Control and Prevention, Beijing, China; ^5^ The George Institute for Global Health, UK, London, United Kingdom; ^6^ School of Public Health, Faculty of Medicine, Imperial College London, London, United Kingdom; ^7^ Centre for Big Data Research in Health, Faculty of Medicine, University of New South Wales, Sydney, NSW, Australia; ^8^ School of Public Health, Harbin Medical University, Harbin, China

**Keywords:** China, accessibility, scoping review, routinely collected health data, record linkage

## Abstract

**Objectives:** The potential for using routinely collected data for medical research in China remains unclear. We sought to conduct a scoping review to systematically characterise nation-wide routinely collected datasets in China that may be of value for clinical research.

**Methods:** We searched public databases and the websites of government agencies, and non-government organizations. We included nation-wide routinely collected databases related to communicable diseases, non-communicable diseases, injuries, and maternal and child health. Database characteristics, including disease area, data custodianship, data volume, frequency of update and accessibility were extracted and summarised.

**Results:** There were 70 databases identified, of which 46 related to communicable diseases, 20 to non-communicable diseases, 1 to injury and 3 to maternal and child health. The data volume varied from below 1000 to over 100,000 records. Over half (64%) of the databases were accessible for medical research mostly comprising communicable diseases.

**Conclusion:** There are large quantities of routinely collected data in China. Challenges to using such data in medical research remain with various accessibility. The potential of routinely collected data may also be applicable to other low- and middle-income countries.

## Introduction

Routinely collected health data (hereafter routinely collected data) is a valuable resource containing large quantities and varieties of information. Routinely collected data are commonly defined as data collected for purposes other than research, such as health service delivery and disease monitoring [1]. Regional and national routine data collections may cover a large proportion of, or entire populations, over extended periods [2]. Common examples include data used to administer health services, disease registries, disease surveillance systems and electronic health records [3]. Such databases are increasingly considered as broad resources with great potential for clinical research, epidemiological studies and health system research [4, 5].

Primary data collection for research has become increasingly resource-intensive and leveraging routinely collected data for research is therefore an attractive and expanding research strategy [6]. Large volumes of data may be accessed in a highly cost-effective way, with many clinical trials, observational studies and health policy and system research around the world using routinely collected data to great effect [7–10]. The use of routinely collected data to assess randomized clinical trial outcomes has been recognized as a disruptive technology for participant recruitment and follow-up [11]. Study participants can be followed at a lower cost and for longer periods to identify long-term effects [12]. In addition, claims data has been used to facilitate pragmatic trials and to do trials embedded within health insurance systems [13, 14].

China has established multiple health databases over the past 2 decades with several examples of these data being used for clinical research—health insurance claims data have been used in a large prospective cohort study [15] and death surveillance data for the identification of fatal outcomes in a large-scale randomized controlled trial [16].

### Objective

The breadth of databases available in China is not, however, defined and the potential for the use of routinely collected data in research is unclear. We conducted this review to identify and characterize databases routinely compiling health information about communicable diseases, non-communicable diseases (NCD), injuries and maternal and child health in China.

## Methods

This review was conducted following an established framework—the Preferred Reporting Items for Systematic Reviews and Meta-analyses Extension for Scoping Reviews (PRISMA-ScR) [17, 18]. This review was registered on Open Science Framework (10.17605/OSF.IO/Q5CNB).

### Search Strategy

We searched in four places for routinely collected health databases. First, on the websites of Chinese government agencies that do work related to health, medicine or data including the Chinese National Health Commission, the Centre for Disease Control and Prevention (CDC), the National Medical Products Administration, the National Bureau of Statistics, the Ministry of Science and Technology, the Ministry of Transport, the Ministry of Public Security and the Ministry of Civil Affairs. Second, on the websites of international institutions collaborating with China on health issues including the Global Burden of Disease (GBD), the World Health Organization, the United Nations International Children’s Emergency Fund and the World Bank. Third, we conducted an internet search using Google and the local Chinese search engine (Baidu) using keywords for disease types based on the International Classification of Disease 10th Revision and the disease classifications of the GBD data (See Supplementary Table S1). Lastly, we searched the published literature in English and Chinese language journals for studies that mentioned routinely collected data in China. The English language databases searched were EMBASE, Medline, Scopus and CENTRAL. The Chinese language databases searched were the Chinese National Knowledge Infrastructure (CNKI) and Wanfang. The maximum extent of the search period was from Jan 1946 to May 2020 and keywords used included “routinely collected data,” “registry” and “surveillance” with full details in supplementary materials (See Supplementary Methods).

### Selection Criteria

Databases were eligible for inclusion if they: 1) had nation-wide coverage of mainland China; 2) contained information about healthcare delivery, health outcomes, treatments or health expenditures; 3) held data related to communicable diseases, NCDs, injuries or maternal and child health; and 4) were ongoing and regularly updated. All potentially eligible databases were reviewed independently by two reviewers (YL and SX) with any inconsistency regarding eligibility resolved through discussion.

### Data Extraction and Synthesis

For all eligible databases, we sought to extract standard information describing the data custodian, purpose, time of establishment, volume of data, update frequency, data collection methods, data fields and accessibility. The data extraction was conducted independently by two reviewers (YL and SX) with consensus achieved through consultation. We summarised the databases characteristics by disease areas (communicable diseases, NCDs, injuries and maternal and child health), the volume of data available by May 2020 (less than 10,000 records; more than 10,000 and less than 100,000 records; more than 100,000 records), accessibility (aggregated data available; individual data available by application; confidential; unknown) and method of access (access online, access by application, unknown).

## Results

**FIGURE 1:**
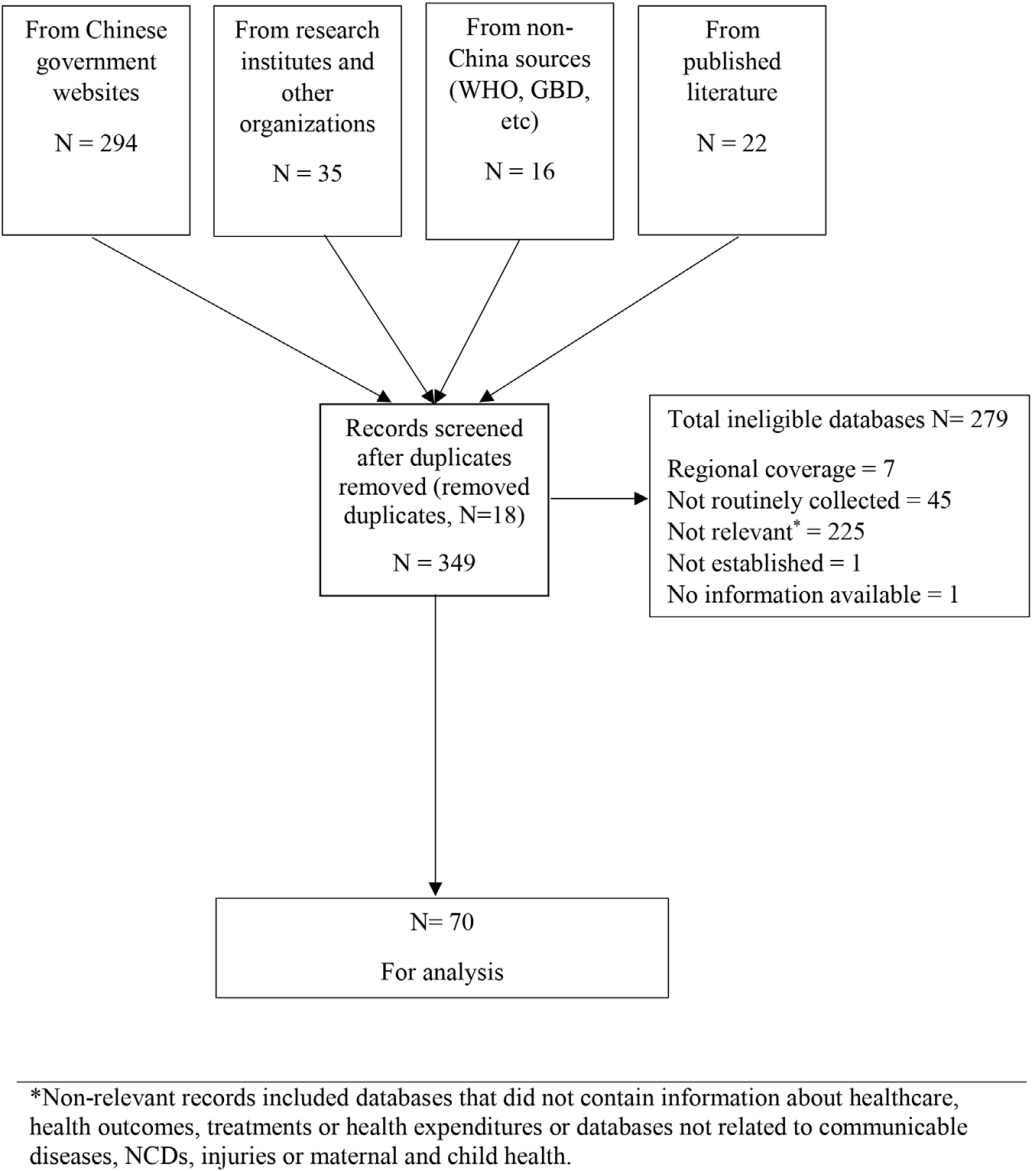
Flow chart showing database search and study selection process (scoping review, China, 1946–2020) *Non-relevant records included databases that did not contain information about healthcare, health outcomes, treatments or health expenditures or databases not related to communicable diseases, NCDs, injuries or maternal and child health.

We identified 349 potentially eligible databases with most from government agency websites. We excluded 279 mostly because they did not address a specified disease area (*n* = 225), were not regularly updated (*n* = 45) or did not have nation-wide coverage (*n* = 7) (Figure 1). Some databases were ineligible for multiple reasons. There were 70 databases finally included (Supplementary Table S2).

### Types and Sources of Routinely Collected Health Data

Routinely collected databases relating to communicable disease (*n* = 46/70, 66%) and NCD (*n* = 20/70, 29%) were the majority identified (Table 1). Among all the databases, 81% (*n* = 57) were used for surveillance purposes and 19% (*n* = 13) were disease registries. Disease surveillance databases mostly covered communicable diseases (46/57) but were also used for birth defects, injuries and maternal and child health. The disease registries only covered NCDs such as stroke, acute myocardial infarction, cancer and some rare diseases. There were no nation-wide health administrative databases identified.

**TABLE 1:** Characteristics of routinely collected databases (scoping review, China, 1946–2020).

	Communicable disease	NCD	Injury	Maternal and child health	Overall
Total number	46	20	1	3	70
Types of database					
Health administrative data	−	−	−	−	−
Surveillance system	46	7	1	3	57
Disease registry	−	13	−	−	13
Year of establishment					
Before 2000	1	2	−	3	6
2000–−2010	45	4	1	−	50
2011–2020	−	14	−	−	14
Custodian					
Government agencies	45	8	1	2	56
Research institutes/universities	1	9	−	1	11
Public hospitals	−	3	−	−	3
Number of data records^a^					
≤10,000	13	3	−	−	17
≥10,000 and ≤100,000	7	−	−	−	7
≥100,000	24	−	−	1	26
Unknown	2	17	1	2	20
Updating frequency					
Real time^b^	6	−	−	−	6
Monthly	36	1	−	−	37
Yearly	1	4	1	2	8
Every 3 years	−	2	−	−	2
Every 5 years	−	2	−	−	2
Unknown	3	11	−	1	15
Accessibility					
Aggregated data available^b^	−	1	−	1	2
Individual data available by application^c^	43	2	−	−	45
Confidential	1	−	−	−	1
Unknown	2	17	1	2	22

a Records refer to individual episode of disease or individual person depending on the types of data.

b Aggregated data available but individual data unknown.

c Aggregated data available online and individual data only available by application to data owners.

The majority of the routinely collected data were under the custodianship of government agencies (*n* = 56/70, 80%) or research institutes (*n* = 11/70, 16%). Almost all routinely collected data related to communicable diseases (45/46) were managed by the China Centres for Disease Control. For NCDs, 8 databases were managed by government agencies, 9 by research institutes and 3 by public hospitals. Three of the four databases holding information on injuries and maternal and child health were managed by government agencies and one by a research institute.

### Establishment of Databases Over Time

**FIGURE 2:**
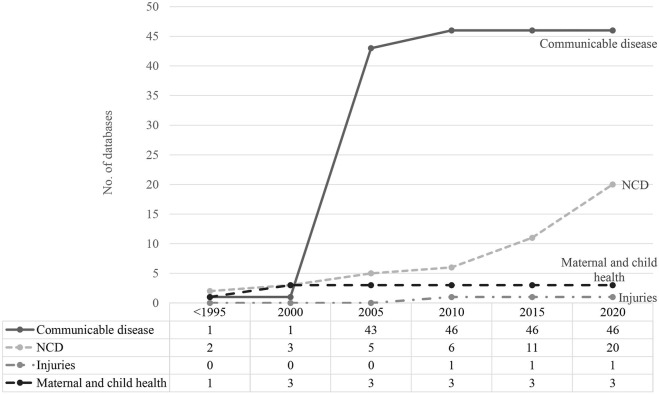
Time trend of routinely collected data development (scoping review, China, 1946–2020) Numbers of routinely collected data related to communicable and non-communicable diseases, injuries and maternal and child health were accumulated since <1995 until 2020.

Prior to 2000, there were few routinely collected databases in any disease category. There was rapid growth in routinely collected data related to communicable diseases after 2000, with 42 new databases established between 2003 and 2005 (Figure 2). Significant expansion in databases recording information about NCDs was not observed until 2015.

### Data Volume and Frequency of Data Updates

Information about the volume of data was available for 47 (67%) databases and information about the frequency of updating for 55 (79%). There were 26 databases (37%) that reported holding data on more than 100,000 individuals and 24 of these were databases related to communicable diseases. For 17 of the databases related to NCDs data volumes were unknown. In general, the databases of communicable diseases were updated more frequently than databases for NCDs, with 36 of the communicable disease surveillance systems updated monthly and 6 implemented as real time reporting systems. Databases related to NCDs, injuries and maternal and child health were updated between once a month and once every 5 years.

### Accessibility of Databases

Information about access to the data was available for 47 (67%) databases. Data of the 45 (64%) databases were readily accessible, mostly comprising communicable disease surveillance data held by the China Centres for Disease Control. For these databases, aggregated data were available online, while individual data can be acquired by application with a potential cost (Table 2). There were two databases that published aggregated data but for which the potential to access individual data was unclear. The accessibility of data related to NCDs (17/20) could mostly not be identified.

**TABLE 2:** Characteristics of included databases by data custodians (scoping review, China, 1946–2020).

	Data custodian		
	Government agencies	Research institutes/universities	Other
Details of owners	Mainly owned by CDC and CDC affiliated institutes, such as National Institute for Communicable Disease Prevention and Control, National Institute for Nutrition and Health etc.	Independent research institutes and university affiliated institutes	Mainly hospitals leading specific disease registries
Types of diseases covered	Majority of communicable diseases and common NCD databases and all databases of injuries and maternal and child health	Disease registries of NCDs	Disease registries of NCDs with relatively lower prevalence such as rare disease registries
Types of databases	Surveillance	Disease registry	Disease registry
Aggregated data availability	Aggregated data available online without application, for most of the CDC owned databases	Only small numbers of databases with aggregated data available online	Mostly unknown availability
Individual data availability	Most of the CDC owned databases are publicly available and individual records are available by application with potential cost if data requires extra processing or data are requested in non-electronic forms; some databases only have aggregated data available and the availability of individual records is unknown	Mostly unknown	Mostly unknown

## Discussion

The majority of accessible routinely collected data in China derive from databases established for the surveillance of communicable diseases and are under the custodianship of government agencies. Much fewer data relating to NCDs and injuries are collected and clearly accessible.

The nature of the routinely collected health data available in China reflects the evolution of public health priorities in the country. The earliest systems established in the 1950s [19] focussed on infectious diseases with rapid expansion after 2003 following the severe acute respiratory syndrome (SARS) epidemic. The Chinese government invested significant resources in infectious disease monitoring at this time, to strengthen established public health systems and implement multiple new surveillance programs [20]. These systems were designed primarily to enable better healthcare provision but also allowed for greatly enhanced research activity and reporting on infectious disease epidemiology [21].

The growth in databases related to NCDs has accelerated in the last decade with the launch of the 2009 health-care reform plan, prompting the development of health information systems focused on chronic conditions [22]. The expanding focus on databases recording information about NCDs in China is clearly warranted by the shift in disease burden from communicable to non-communicable diseases that has occurred [23, 24]. However, this review identified only limited (less than 30%) routinely collected databases related to NCDs. The NCD monitoring has to rely on the national health surveys conducted every few years [25]. The monitoring of NCD burdens and healthcare services will hence be limited and delayed. The key challenge of establishing routinely collected NCD data is the multiple source data custodianships making it hard to timely integrate the data. At the same time, the SARS-Coronavirus 2 pandemic has posed new challenges to infectious diseases surveillance systems [26] and there are likely to be multiple new communicable disease databases as a consequence of the pandemic. Novel surveillance methods based on space-time tracing technologies, syndromic surveillance systems and citywide pandemic monitoring platforms have been developed to combat the SARS-Coronavirus 2 pandemic in China, as they have in many other countries around the world [27, 28].

There were no Chinese electronic health record systems identified as eligible for inclusion in the review. This was mainly because all operate at a sub-national level with most patient data, and similarly, health insurance claims data held and managed by individual hospitals of regional administrative bodies. In other Asian countries such as Japan and Malaysia, integrated health information systems have been established by the Ministry of Health to link patient data from individual hospitals, mostly from public hospitals representing more than half of the inpatient admissions in the country [29]. The infrastructures of the existing information systems can serve as the cornerstones to achieving complete population-wide coverage in the future. In a few countries such as the UK, Canada and Australia, data from these systems have been widely used for research purposes, illustrating their enormous potential [30–32]. The National Health Service in the UK, for example, provides access to nation-wide data about primary care consultations and hospital admissions that have been used for studies of disease incidence [33], health service performance [34], medicine prescription patterns [35], as well as to collect outcomes for clinical trials of therapeutic interventions [36]. In regard to the latter, routinely collected data may save considerable resources compared to traditional data collection methods, and has been used in China for this purpose [15, 37], though issues with data quality and completeness have been identified [16]. A key challenge is that the investment in the curation of routinely collected data is typically not as high as might be made for a standalone research project, and the data may be more prone to both systemic and random errors as a consequence [38]. In addition, the infrastructure required to achieve timely data-sharing agreements with data custodians is limited in China, as it is elsewhere around the world [32], and there are significant investments required to implement the technical solutions and operating protocols required to enable data manipulation while ensuring data security.

Provided the large quantities of existing routinely collected databases with nation-wide coverage in China, there has been great potential for such data to be applied in large medical research. The China Kadoorie Biobank follows half a million participants by linking to the routinely collected health insurance claims data to identify disease occurrences [15]. A growing number of large cohort studies in China have used record linkage to routinely collected health data such as health insurance claims, health administrative data and mortality surveillance to follow up study participants over the long run [39–41]. The linkage to health insurance claims data has also been successfully applied in the large randomized controlled trial conducted in China [37]. Likewise, in other low and middle income countries with nation-wide routinely collected health datasets, there may be significant potential to apply these data in high quality medical research.

### Strength and Limitations

This review benefits from the extensive searches of databases done in Chinese and English languages and the standardised extraction and processing of the data by independent reviewers. The exclusion of data collections done at the sub-national level, by provinces or cities, means that the quantum of routinely collected health data in China has likely been significantly underestimated, though the challenges in accessing tens to hundreds of individual databases to do a national study would be enormous. We were also not able to extract information about the completeness or quality of the data held in each repository and the utility of the routinely collected data may be good for some types of research studies but inadequate for others. Missing data about aspects of multiple of the identified databases represented a significant challenge too.

### Conclusion

There are large national databases in China that offer significant opportunities for researchers addressing communicable diseases but routinely collected data describing non-communicable diseases and injuries, the leading national causes of disease burden, are currently limited. The significant national investment in collecting routine health data warrants further exploration of the potential for using these data for health research and similarly in other low- and middle-income countries. There will, however, need to be substantial coordination of activities regarding data collection, security, management and sharing, across the national departments and institutes including the Ministry of Health, Finance, Statistics and other relevant sectors to reap the full research potential from the data that are held [42, 43].

## Abbreviations

NCD, noncommunicable disease; CDC, center for disease control and prevention; GBD, global burden of disease; SARS, severe acute respiratory syndrome.
