# Supplementary material for: Nation-Wide Routinely Collected Health Datasets in China: A Scoping Review
Source: Public Health Rev. 2022 Sep 21;43:1605025. doi: 10.3389/phrs.2022.1605025 (PMC9532513; doi:10.3389/phrs.2022.1605025)
Supplement: Supplementary file 1 [file DataSheet1.docx]

**Supplementary materials**

1. Table S1: list of keywords for disease types
2. Methods: list of search keywords
3. Table S2: detailed information on the included databases

**Table S1: list of all the disease keywords for search**

| Communicable diseases and maternal and child health | Disease Category | | |
| --- | --- | --- | --- |
| A. | Infectious and parasitic diseases | | |
|  | 1. | Tuberculosis | |
|  | 2. | STDs excluding HIV | |
|  |  | a. | Syphilis |
|  |  | b. | Chlamydia |
|  |  | c. | Gonorrhoea |
|  |  | d. | Trichomoniasis |
|  |  | e. | Genital herpes |
|  |  | f. | Other STDs |
|  | 3. | HIV/AIDS |  |
|  | 4. | Diarrhoeal diseases | |
|  | 5. | Childhood-cluster diseases | |
|  |  | a. | Whooping cough |
|  |  | b. | Diphtheria |
|  |  | c. | Measles |
|  |  | d. | Tetanus |
|  | 6. | Meningitis |  |
|  | 7. | Encephalitis | |
|  | 8. | Hepatitis |  |
|  |  | a. | Acute hepatitis A |
|  |  | b. | Acute hepatitis B |
|  |  | c. | Acute hepatitis C |
|  |  | d. | Acute hepatitis E |
|  | 9. | Parasitic and vector diseases | |
|  |  | a. | Malaria |
|  |  | b. | African Trypanosomiasis |
|  |  | c. | Chagas disease |
|  |  | d. | Schistosomiasis |
|  |  | e. | Leishmaniasis |
|  |  | f. | lymphatic filariasis |
|  |  | g. | Onchocerciasis |
|  |  | h. | Cysticercosis |
|  |  | i. | Echinococcosis |
|  |  | j. | Dengue |
|  |  | k. | Trachoma |
|  |  | l. | Yellow fever |
|  |  | m. | Rabies |
|  | 10. | Intestinal nematode infections | |
|  |  | a. | Ascariasis |
|  |  | b. | Trichuriasis |
|  |  | c. | Hookworm disease |
|  |  | d. | Food-bourne trematodes |
|  | 11. | Leprosy |  |
|  | 12. | Other infectious diseases | |
| B. | Respiratory Infectious | | |
|  | 1. | Lower respiratory infections | |
|  | 2. | Upper respiratory infections | |
|  | 3. | Otitis media | |
| C. | Maternal conditions | |  |
| D. | Neonatal conditions | |  |
|  | 1. | Preterm birth complications | |
|  | 2. | Birth asphyxia and birth trauma | |
|  | 3. | Neonatal sepsis and infections | |
|  | 4. | Other neonatal conditions | |
| E. | Nutritional deficiencies | | |
|  | 1. | Protein-energy malnutrition | |
|  | 2. | Iodine deficiency | |
|  | 3. | Vitamin A deficiency | |
|  | 4. | Iron-deficiency anaemia | |
|  | 5. | Other nutritional deficiencies | |
| NCDs |  | | |
| A. | Malignant neoplasms | |  |
|  | 1. | Mouth and oropharynx cancers | |
|  |  | a. | Lip and oral cavity |
|  |  | b. | Nasopharynx |
|  |  | c. | Other pharynx |
|  | 2. | Oesophagus cancer | |
|  | 3. | Stomach cancer | |
|  | 4. | Colon and rectum cancers | |
|  | 5. | Liver cancer | |
|  |  | a. | Liver cancer secondary to hepatitis B |
|  |  | b. | Liver cancer secondary to hepatitis C |
|  |  | c. | Liver cancer secondary to alcohol use |
|  |  | d. | Other liver cancer |
|  | 6. | Pancreas cancer | |
|  | 7. | Trachea, bronchus, lung cancers | |
|  | 8. | Melanoma and other skin cancers | |
|  |  | a. | Malignant skin melanoma |
|  |  | b. | Non-melanoma skin cancer |
|  | 9. | Breast cancer | |
|  | 10. | Cervix uteri cancer | |
|  | 11. | Corpus uteri cancer | |
|  | 12. | Ovary cancer | |
|  | 13. | Prostate cancer | |
|  | 14. | Testicular cancer | |
|  | 15. | Kidney cancer | |
|  | 16. | Bladder cancer | |
|  | 17. | Brain and nervous system cancers | |
|  | 18. | Gallbladder and biliary tract cancer | |
|  | 19. | Larynx cancer | |
|  | 20. | Thyroid cancer | |
|  | 21. | Mesothelioma | |
|  | 22. | Lymphomas, multiple myeloma | |
|  |  | a. | Hodgkin lymphoma |
|  |  | b. | Non-Hodgkin lymphoma |
|  |  | c. | Multiple myeloma |
|  | 23. | Leukaemia | |
|  | 24. | Other malignant neoplasms | |
| B. | Other neoplasms | |  |
| C. | Diabetes mellitus | |  |
| D. | Endocrine, blood, immune disorders | | |
|  | 1. | Thalassaemias | |
|  | 2. | Sickle cell disorders and trait | |
|  | 3. | Other haemoglobinopathies and haemolytic anaemias | |
|  | 4. | Other endocrine, blood and immune disorders | |
| E. | Mental and substance use disorders | | |
|  | 1. | Depressive disorders | |
|  |  | a. | Major depressive disorder |
|  |  | b. | Dysthymia |
|  | 2. | Bipolar disorder | |
|  | 3. | Schizophrenia | |
|  | 4. | Alcohol use disorders | |
|  | 5. | Drug use disorders | |
|  |  | a. | Opioid use disorders |
|  |  | b. | Cocaine use disorders |
|  |  | c. | Amphetamine use disorders |
|  |  | d. | Cannabis use disorders |
|  |  | e. | Other drug use disorders |
|  | 6. | Anxiety disorders | |
|  | 7. | Eating disorders | |
|  | 8. | Autism and Asperger syndrome | |
|  | 9. | Childhood behavioural disorders | |
|  |  | a. | Attention deficit/hyperactivity syndrome |
|  |  | b. | Conduct disorder |
|  | 10. | Idiopathic intellectual disability | |
|  | 11. | Other mental and behavioural disorders | |
| F. | Neurological conditions | | |
|  | 1. | Alzheimer disease and other dementias | |
|  | 2. | Parkinson disease | |
|  | 3. | Epilepsy |  |
|  | 4. | Multiple sclerosis | |
|  | 5. | Migraine |  |
|  | 6. | Non-migraine headache | |
|  | 7. | Other neurological conditions | |
| G. | Sense organ diseases | | |
|  | 1. | Glaucoma |  |
|  | 2. | Cataracts |  |
|  | 3. | Uncorrected refractive errors | |
|  | 4. | Macular degeneration | |
|  | 5. | Other vision loss | |
|  | 6. | Other hearing loss | |
|  | 7. | Other sense organ disorders | |
| H. | Cardiovascular diseases | | |
|  | 1. | Rheumatic heart disease | |
|  | 2. | Hypertensive heart disease | |
|  | 3. | Ischaemic heart disease | |
|  | 4. | Stroke |  |
|  |  | a. | Ischaemic stroke |
|  |  | b. | Haemorrhagic stroke |
|  | 5. | Cardiomyopathy, myocarditis, endocarditis | |
|  | 5. | Cardiomyopathy, myocarditis, endocarditis | |
|  | 6. | Other circulatory diseases | |
| I. | Respiratory diseases | |  |
|  | 1. | Chronic obstructive pulmonary disease | |
|  | 2. | Asthma |  |
|  | 3. | Other respiratory diseases | |
| J. | Digestive diseases | |  |
|  | 1. | Peptic ulcer disease | |
|  | 2. | Cirrhosis of the liver | |
|  |  | a. | Cirrhosis due to hepatitis B |
|  |  | b. | Cirrhosis due to hepatitis C |
|  |  | c. | Cirrhosis due to alcohol use |
|  |  | d. | Other liver cirrhosis |
|  | 3. | Appendicitis | |
|  | 4. | Gastritis and duodenitis | |
|  | 5. | Paralytic ileus and intestinal obstruction | |
|  | 6. | Inflammatory bowel disease | |
|  | 7. | Gallbladder and biliary diseases | |
|  | 8. | Pancreatitis | |
|  | 9. | Other digestive diseases | |
| K. | Genitourinary diseases | | |
|  | 1. | Kidney diseases | |
|  |  | a. | Acute glomerulonephritis |
|  |  | b. | Chronic kidney disease due to diabetes |
|  |  | c. | Other chronic kidney disease |
|  | 2. | Benign prostatic hyperplasia | |
|  | 3. | Urolithiasis | |
|  | 4. | Other urinary diseases | |
|  | 5. | Infertility |  |
|  | 6. | Gynecological diseases | |
| L. | Skin diseases | |  |
| M. | Musculoskeletal diseases | | |
|  | 1. | Rheumatoid arthritis | |
|  | 2. | Osteoarthritis | |
|  | 3. | Gout |  |
|  | 4. | Back and neck pain | |
|  | 5. | Other musculoskeletal disorders | |
| N. | Congenital anomalies | | |
|  | 1. | Neural tube defects | |
|  | 2. | Cleft lip and cleft palate | |
|  | 3. | Down syndrome | |
|  | 4. | Congenital heart anomalies | |
|  | 5. | Other chromosomal anomalies | |
|  | 6. | Other congenital anomalies | |
| O. | Oral conditions | |  |
|  | 1. | Dental caries | |
|  | 2. | Periodontal disease | |
|  | 3. | Edentulism | |
|  | 4. | Other oral disorders | |
| P. | Sudden infant death syndrome | | |
| Injuries |  | | |
| A. | Unintentional injuries | |  |
|  | 1. | Road injury | |
|  | 2. | Poisonings | |
|  | 3. | Falls |  |
|  | 4. | Fire, heat and hot substances | |
|  | 5. | Drowning |  |
|  | 6. | Exposure to mechanical forces | |
|  | 7. | Natural disasters | |
|  | 8. | Other unintentional injuries | |
| B. | Intentional injuries | |  |
|  | 1. | Self-harm |  |
|  | 2. | Interpersonal violence | |
|  | 3. | Collective violence and legal intervention | |

**Methods**

List of search keywords for routinely collected health data related literature.

English keyword search: “routinely collected data*”/”routinely collected health data*”/”routinely collected adj2 data*”/”routinely collected adj3 data*”/”routine data*”/”routine health data*”/”routine adj2 data*”/”registry”/”registries”/”register”/”registration”/”surveillance”/”national adj2 data*/

Chinese keyword search: “数据库”/”大数据“/”数据集“/“疾病注册”/“疾病登记”/“登记系统”/“注册系统”/“疾病监测”/“疾病监控”/“监测”/

**Table S2: list of included databases**

| **Name** | **disease/ condition type** | **database type** | **description** | **start year** | **data update frequency** | **data volume** | **institute owing the database** | **availability** |
| --- | --- | --- | --- | --- | --- | --- | --- | --- |
| Typhoid surveillance | CD | disease surveillance | Contains demographic information and individual episodes of patients, summarized disease incidence and mortality rate. | 2004 | monthly | 10000 /year | CDC | Aggregated data available online, individual data available by application with potential fees |
| Cholera surveillance | CD | disease surveillance | Contains demographic information and individual episodes of patients, summarized disease incidence and mortality rate. | 2004 | monthly | 10/year | CDC | Aggregated data available online, individual data available by application with potential fees |
| Polio surveillance | CD | disease surveillance | Contains demographic information and individual episodes of patients, summarized disease incidence and mortality rate. | 2004 | monthly | 1/year | CDC | Aggregated data available online, individual data available by application with potential fees |
| Hepatitis surveillance | CD | disease surveillance | Contains demographic information and individual episodes of patients, summarized disease incidence and mortality rate. | 2004 | monthly | 120000/year | CDC | Aggregated data available online, individual data available by application with potential fees |
| Dysentery surveillance | CD | disease surveillance | Contains demographic information and individual episodes of patients, summarized disease incidence and mortality rate. | 2004 | monthly | 100000/year | CDC | Aggregated data available online, individual data available by application with potential fees |
| Pertussis surveillance | CD | disease surveillance | Contains demographic information and individual episodes of patients, summarized disease incidence and mortality rate. | 2004 | monthly | 10000/year | CDC | Aggregated data available online, individual data available by application with potential fees |
| Scarlet fever surveillance | CD | disease surveillance | Contains demographic information and individual episodes of patients, summarized disease incidence and mortality rate. | 2004 | monthly | 70000/year | CDC | Aggregated data available online, individual data available by application with potential fees |
| Epidemic meningitis surveillance | CD | disease surveillance | Contains demographic information and individual episodes of patients, summarized disease incidence and mortality rate. | 2004 | monthly | 100/year | CDC | Aggregated data available online, individual data available by application with potential fees |
| SARS surveillance | CD | disease surveillance | Contains demographic information and individual episodes of patients, summarized disease incidence and mortality rate. | 2004 | real time | 10 in 2004 | CDC | Aggregated data available online, individual data available by application with potential fees |
| Influenza A virus subtype H1N1 surveillance | CD | disease surveillance | Contains demographic information and individual episodes of patients, summarized disease incidence and mortality rate. | 2009 | monthly | 147337 in 2009, 7123 in 2010,9360 in 2011,1072 in 2012, 10841 in 2013, 0 after | CDC | Aggregated data available online, individual data available by application with potential fees |
| Diphtheria surveillance | CD | disease surveillance | Contains demographic information and individual episodes of patients, summarized disease incidence and mortality rate. | 2004 | monthly | unknown | CDC | Aggregated data available online, individual data available by application with potential fees |
| Measles surveillance | CD | disease surveillance | Contains demographic information and individual episodes of patients, summarized disease incidence and mortality rate. | 2004 | monthly | before 2010: 10000-100000/year after 2010: <50000/year | CDC | Aggregated data available online, individual data available by application with potential fees |
| Pulmonary tuberculosis surveillance | CD | disease surveillance | Contains demographic information and individual episodes of patients, summarized disease incidence and mortality rate. | 2004 | monthly | 800000/year | CDC | Aggregated data available online, individual data available by application with potential fees |
| Tuberculosis management and reporting system | CD | disease surveillance | Contains demographic information and individual episodes of patients, summarized disease incidence and mortality rate. | 2005 | real time | unknown | CDC | unknown |
| Plague surveillance | CD | disease surveillance | Contains demographic information and individual episodes of patients, summarized disease incidence and mortality rate. | 2004 | real time | 1/year | CDC | Aggregated data available online, individual data available by application with potential fees |
| Anthrax surveillance | CD | disease surveillance | Contains demographic information and individual episodes of patients, summarized disease incidence and mortality rate. | 2004 | monthly | 300/year | CDC | Aggregated data available online, individual data available by application with potential fees |
| Schistosomiasis surveillance | CD | disease surveillance | Contains demographic information and individual episodes of patients, summarized disease incidence and mortality rate. | 2004 | monthly | 2000-5000/year, 34143 in 2015 | CDC | Aggregated data available online, individual data available by application with potential fees |
| Hemorrhagic fever surveillance | CD | disease surveillance | Contains demographic information and individual episodes of patients, summarized disease incidence and mortality rate. | 2004 | monthly | 10000/year | CDC | Aggregated data available online, individual data available by application with potential fees |
| Leptospirosis surveillance | CD | disease surveillance | Contains demographic information and individual episodes of patients, summarized disease incidence and mortality rate. | 2004 | monthly | 100-1000/year | CDC | Aggregated data available online, individual data available by application with potential fees |
| Malaria surveillance | CD | disease surveillance | Contains demographic information and individual episodes of patients, summarized disease incidence and mortality rate. | 2004 | real time | before 2010:10000-100000/year, after 2010:3000-4000/year | CDC | Aggregated data available online, individual data available by application with potential fees |
| Rabies surveillance | CD | disease surveillance | Contains demographic information and individual episodes of patients, summarized disease incidence and mortality rate. | 2004 | monthly | 500-5000/year | CDC | Aggregated data available online, individual data available by application with potential fees |
| Brucellosis surveillance | CD | disease surveillance | Contains demographic information and individual episodes of patients, summarized disease incidence and mortality rate. | 2004 | monthly | 30000-40000/year | CDC | Aggregated data available online, individual data available by application with potential fees |
| Avian influenza (in human) surveillance | CD | disease surveillance | Contains demographic information and individual episodes of patients, summarized disease incidence and mortality rate. | 2004 | real time | 1-10/year | CDC | Aggregated data available online, individual data available by application with potential fees |
| Epidemic encephalitis B | CD | disease surveillance | Contains demographic information and individual episodes of patients, summarized disease incidence and mortality rate. | 2004 | monthly | 1000-10000/year | CDC | Aggregated data available online, individual data available by application with potential fees |
| Dengue surveillance | CD | disease surveillance | Contains demographic information and individual episodes of patients, summarized disease incidence and mortality rate. | 2004 | monthly | <5000/year, 46864 in 2014 | CDC | Aggregated data available online, individual data available by application with potential fees |
| Syphilis surveillance | CD | disease surveillance | Contains demographic information and individual episodes of patients, summarized disease incidence and mortality rate. | 2004 | monthly | 100000-500000/year | CDC | Aggregated data available online, individual data available by application with potential fees |
| Gonorrhea surveillance | CD | disease surveillance | Contains demographic information and individual episodes of patients, summarized disease incidence and mortality rate. | 2004 | monthly | 100000-200000/year | CDC | Aggregated data available online, individual data available by application with potential fees |
| AIDS surveillance | CD | disease surveillance | Contains demographic information and individual episodes of patients, summarized disease incidence and mortality rate. | 2005 | real time | 30000-150000/year | CDC | Aggregated data available online, individual data available by application with potential fees |
| Neonatal tetanus surveillance | CD | disease surveillance | Contains demographic information and individual episodes of patients, summarized disease incidence and mortality rate. | 2004 | monthly | 10-3000/year | CDC | Aggregated data available online, individual data available by application with potential fees |
| Visceral leishmaniasis surveillance | CD | disease surveillance | Contains demographic information and individual episodes of patients, summarized disease incidence and mortality rate. | 2004 | monthly | 100-500/year | CDC | Aggregated data available online, individual data available by application with potential fees |
| Schistosomiasis surveillance | CD | disease surveillance | Contains demographic information and individual episodes of patients, summarized disease incidence and mortality rate. | 2004 | monthly | 320 | CDC | Aggregated data available online, individual data available by application with potential fees |
| Rubella surveillance | CD | disease surveillance | Contains demographic information and individual episodes of patients, summarized disease incidence and mortality rate. | 2004 | monthly | 10000-100000/year before 2015, <10000 after 2015 | CDC | Aggregated data available online, individual data available by application with potential fees |
| Mumps surveillance | CD | disease surveillance | Contains demographic information and individual episodes of patients, summarized disease incidence and mortality rate. | 2004 | monthly | 200000-300000/year | CDC | Aggregated data available online, individual data available by application with potential fees |
| Other infectious diarrhea surveillance | CD | disease surveillance | Contains demographic information and individual episodes of patients, summarized disease incidence and mortality rate. | 2004 | monthly | 400000-1200000/year | CDC | Aggregated data available online, individual data available by application with potential fees |
| Hydatid disease surveillance | CD | disease surveillance | Contains demographic information and individual episodes of patients, summarized disease incidence and mortality rate. | 2004 | monthly | 600-5000/year | CDC | Aggregated data available online, individual data available by application with potential fees |
| Typhus surveillance | CD | disease surveillance | Contains demographic information and individual episodes of patients, summarized disease incidence and mortality rate. | 2004 | monthly | 900-4000/year | CDC | Aggregated data available online, individual data available by application with potential fees |
| Acute hemorrhagic conjunctivitis surveillance | CD | disease surveillance | Contains demographic information and individual episodes of patients, summarized disease incidence and mortality rate. | 2004 | monthly | 10000-70000/year | CDC | Aggregated data available online, individual data available by application with potential fees |
| Filariasis surveillance | CD | disease surveillance | Contains demographic information and individual episodes of patients, summarized disease incidence and mortality rate. | 2004 | monthly | <10/year | CDC | Aggregated data available online, individual data available by application with potential fees |
| Hand, foot, and mouth disease surveillance | CD | disease surveillance | Contains demographic information and individual episodes of patients, summarized disease incidence and mortality rate. | 2008 | monthly | 500000-3000000/year | CDC | Aggregated data available online, individual data available by application with potential fees |
| Influenza surveillance | CD | disease surveillance | Contains demographic information and individual episodes of patients, summarized disease incidence and mortality rate. | 2004 | monthly | 50000-500000/year | CDC | Aggregated data available online, individual data available by application with potential fees |
| Leprosy surveillance | CD | disease surveillance | Contains demographic information and individual episodes of patients, summarized disease incidence and mortality rate. | 2004 | monthly | 300-400/year | CDC | Aggregated data available online, individual data available by application with potential fees |
| Chinese nutrition and health surveillance | NCD/  Nutrition | surveillance data | Contains information on food and nutrition intakes, behaviours and lifestyle factors, and nutrition related biomarkers such as blood lipids profile and blood pressure. | 1959 | unknown | unknown | CDC | Aggregated data available online, individual data available by application with potential fees |
| The Youth Risk Behavior Surveillance System | Adolescent health | surveillance data | Contains information on health risk factors related to behaviours among teenagers. | 1998 | unknown | 224000 | Peking University | unknown |
| Disease surveillance points system (DSP) Internet-based reporting system | Mortality | death surveillance | Contains common disease surveillance information from 2006 (internet-bases system). | 1978 | yearly | unknown | CDC | unknown |
| Infectious disease internet-based reporting system, demographic database | CD | disease surveillance | Contains demographic information and individual episodes of patients, summarized disease incidence and mortality rate. | 2004 | yearly | 1300000000 | CDC | Aggregated data available online, individual data available by application with potential fees |
| Infectious disease origins surveillance | CD | disease surveillance | Contains demographic information and individual episodes of patients, summarized disease incidence and mortality rate. | 1990 | unknown | 150000 | Academy of Military Medical Sciences | Aggregated data available online, individual data available by application with potential fees |
| Infectious disease surveillance | CD | disease surveillance | Contains demographic information and individual episodes of patients, summarized disease incidence and mortality rate. | 2004 | monthly | 8387334142 | CDC | Aggregated data available online, individual data available by application with potential fees |
| Maternal and child health surveillance system (MCHS) | Maternal and Child Health | surveillance data | Contains surveillance data on death under age 5, maternal death, birth defect. | 1996 | yearly | unknown | Ministry of Health | unknown |
| National surveillance of physique | NCD/General Health | surveillance data | Contains information on demographic data, health risk factors such as physical activities, behaviours, lifestyles, assessment of physique, etc. | 2000 | every 5 years | unknown | China Institute of Sport Science | unknown |
| Chinese NCD and NCD risk factors surveillance | NCD | surveillance data | Contains surveillance data on anthropometric measurements, lifestyle risk factors. | 2004 | every three years | unknown | CDC | unknown |
| Chinese adult NCD and nutrition surveillance | NCD/Nutrition | surveillance data | Contains surveillance data on anthropometric measurements, nutritional risk factors and NCD related biomarker measurements. | 2015 | every three years | unknown | CDC | unknown |
| National injury surveillance | injury | surveillance data | Contains hospital based injury reporting data, included individual cases of injuries. | 2006 | yearly | unknown | CDC | unknown |
| National chronic obstructive pulmonary disease surveillance | NCD | surveillance data | Contains surveillance data on COPD aged over 40, included disease incidence, and related risk factors. | 2014 | every five years | unknown | CDC | unknown |
| National soil-origined nematode disease surveillance | CD | disease surveillance | Contains demographic information and individual episodes of patients, summarized disease incidence and mortality rate. | 2006 | unknown | unknown | CDC | unknown |
| Mortality surveillance system (integrated DSP and vital registration) | Mortality | surveillance data | National mortality surveillance system, included and integrated data from DSP and vital registration, contains detailed information on death certificate filled by health professionals. | 2013 | yearly | unknown | CDC | Aggregated data available online, individual data available by application with potential fees |
| National Central Cancer Registry | NCD | disease registry | Central cancer registry contains information from 501 local registries for all types of cancers. | 2002 | yearly | unknwon | National Cancer Center | unknown, annual report available |
| Chinese advanced pancreatic cancer registry | NCD | disease registry | Contains demographic information of individual patients, and disease episodes, treatment and follow ups. | 2019 | monthly | unknown | Chinese Society of Clinical Oncology | unknown |
| National brain tumor registry | NCD | disease registry | Contains information on individual episodes of brain tumours and treatment. | 2018 | unknown | unknown | China National Clinical Research Center for Neurological Diseases | unknown(web: https://www.nbtrc.cn/public/login (May 17, 2020), username password needed) |
| National childhood leukemia registry | NCD | disease registry | The first nation-wide disease registry for children. Contains information on demographics, disease episodes, treatment, follow ups and medical costs. | 2018 | unknown | unknown | Shanghai Children's Medical Center; Shanghai Institute of Medical Quality | unknown |
| China early gastrointestinal cancer registry | NCD | disease registry | Contains data on individual episodes of gastrointestinal cancers, treatment, and follow up. | 2017 | unknown | unknown | National Clinical Research Center for Digestive Diseases | unknown |
| National autism registry | NCD | disease registry | Contains data on autism registry, including individual cases, diagnosis, and treatment. | 2019 | unknown | unknown | National Health Commission | unknown |
| Chinese Familial Alzheimer's Disease Network | NCD | disease registry | Contains data on individual episodes of familial Alzheimer’s diseases and treatment. | 2014 | unknown | unknown | Xuanwu Hospital Capital Medical University | unknown |
| Chinese familial Parkinson's disease registry | NCD | disease registry | Contains data on individual episodes of familial Parkinson’s disease and treatments. | 2018 | unknown | 772 | National Research Center of Geriatric Diseases | unknown |
| Chinese early-onset Parkinson's disease registry | NCD | disease registry | Contains data on individual episodes of early-onset Parkinson’s disease and treatments. | 2018 | unknown | 2498 | National Research Center of Geriatric Diseases | unknown |
| China National Stroke Registry | NCD | disease registry | Contains data on individual episodes of strokes registered from secondary and tertiary hospitals. | 2007 | yearly/every four year | unknown | China National Clinical Research Center for Neurological Diseases | unknown |
| Chinese acute myocardial infarction registry | NCD | disease registry | Contains data on individual episodes of AMI registered from hospitals. | 2013 | unknown | unknown | Fuwai Hospital Chinese Academy of Medical Sciences | unknown |
| Chinese Registry of Rheumatoid Arthritis | NCD | disease registry | Contains data on Contains data on individual episodes of rheumatoid diseases and treatment. | 2016 | unknown | 8071 | Chinese Rheumatism Data Center | unknown |
| Chinese rare disease registry | NCD | disease registry | Contains data on individual episodes of rare diseases. | 2016 | unknown | unknown | Peking Union Medical College Hospital | unknown |
| Birth defects surveillance | Maternal and Child Health | surveillance data | Registered all the birht defects reporting from individual hospitals nation-wide | 1986 | yearly | unknown | Ministry of Health | unknown, annual report |
| Public health emergency surveillance | CD | surveillance data | Record all the publich health emergency events | 2004 | unknown | 8174 | CDC | confidential |
